# Construction of ferritin hydrogels utilizing subunit–subunit interactions

**DOI:** 10.1371/journal.pone.0259052

**Published:** 2021-11-03

**Authors:** Masaru Yamanaka, Tsuyoshi Mashima, Michio Ogihara, Mei Okamoto, Takayuki Uchihashi, Shun Hirota

**Affiliations:** 1 Division of Materials Science, Graduate School of Science and Technology, Nara Institute of Science and Technology, Ikoma, Japan; 2 Department of Physics, Nagoya University, Nagoya, Japan; Russian Academy of Medical Sciences, RUSSIAN FEDERATION

## Abstract

Various proteins form nanostructures exhibiting unique functions, making them attractive as next-generation materials. Ferritin is a hollow spherical protein that incorporates iron ions. Here, we found that hydrogels are simply formed from concentrated apoferritin solutions by acid denaturation and subsequent neutralization. The water content of the hydrogel was approximately 80%. The apoferritin hydrogel did not decompose in the presence of 1 M HCl, 2-mercaptoethanol, or methanol but was dissolved in the presence of 1 M NaOH, by heating at 80°C, or by treatment with trypsin or 6 M guanidine hydrochloride. The Young’s modulus of the hydrogel was 20.4 ± 12.1 kPa according to local indentation experimentes using atomic force microscopy, indicating that the hydrogel was relatively stiff. Transition electron microscopy measurements revealed that a fibrous network was constructed in the hydrogel. The color of the hydrogel became yellow-brown upon incubation in the presence of Fe^3+^ ions, indicating that the hydrogel adsorbed the Fe^3+^ ions. The yellow-brown color of the Fe^3+^-adsorbed hydrogel did not change upon incubation in pure water, whereas it became pale by incubating it in the presence of 100 mM ethylenediaminetetraacetic acid (EDTA). The apoferritin hydrogel also adsorbed Co^2+^ and Cu^2+^ ions and released them in the presence of EDTA, while it adsorbed less Ni^2+^ ions; more Fe^3+^ ions adsorbed to the apoferritin hydrogel than other metal ions, indicating that the hydrogel keeps the iron storage characteristic of ferritin. These results demonstrate a new property of ferritin: the ability to form a hydrogel that can adsorb/desorb metal ions, which may be useful in designing future biomaterials.

## Introduction

Various proteins form nanostructures with unique functions, making them attractive as next-generation materials for various fields, including medicine and industry. Natural protein nanostructures have been applied to biomineralization, semiconductor production, gene transfer vectors, and drug delivery systems [1–4], whereas artificial protein nanostructures have been constructed for improved and novel functions [5–23]. Protein hydrogels are an improved functional material that can be obtained by accumulating and immobilizing proteins with various methods [24–27]. Functions have been added to collagen and elastin-like protein hydrogels by chemical modification [28–30]. Functional protein hydrogels have also been constructed by ligation of a protein with a polymer [31] or by self-assembling a coiled-coil protein motif [32].

Ferritin is an iron storage protein, which is widely present in prokaryotes and eukaryotes [1, 3]. Ferritin forms a 12-nm diameter hollow spherical structure consisting of 24 subunits [1, 33, 34]. The intact hollow spherical ferritin core is stable over the pH range 2.1–10.0 according to small-angle X-ray scattering measurements, whereas the ferritin subunits undergo aggregation below pH 0.8 [35]. The spherical ferritin structure only recovers to a headset-shaped structure from disassembled rod-like oligomers by restoring the pH condition to neutral from pH 1.96 [35]. Ferritin incorporates approximately 4500 iron atoms in its interior space [1], as well as cobalt, copper, and other transition metal ions [36–41]. Due to its hollow spherical structure and/or metal-binding properties, ferritin is one of the most promising candidates for future functional materials, such as drug delivery systems, cancer treatment, and memory devices [42–46].

The function of a protein is usually associated with the three-dimensional structure of the protein. When the protein loses its three-dimensional structure in hydrogels, it may lose its function. Functional protein hydrogels have been constructed with three-dimensional proteins utilizing chemical modification or polymer attachment. However, protein hydrogels comprising only proteins are ideal biocompatible materials; thus, we envisaged to construct an apoferritin (ferritin without metal coordination) hydrogel utilizing the subunit interactions, maintaining the three-dimensional structure of the subunit in the hydrogel. The hydrogel appeared to be formed by a network of incomplete intermolecular interaction among ferritin subunits. The apoferritin hydrogel was heat stable and resistant to acidic pH conditions and a reducing agent. The hydrogel also exhibited metal ion adsorption/desorption properties, similar to ferritin.

## Materials and methods

### Preparation of recombinant horse apoferritin and its hydrogel

Recombinant horse ferritin was expressed as reported previously [47]. *Escherichia* (*E*.) *coli* Nova blue cells (Novagen, USA) containing plasmid pKIT8, encoding the gene of horse L apoferritin without eight N-terminal residues (Fer8), were grown in LB broth at 37°C for 24 h. The cells were harvested by centrifugation, and suspended in 50 mM Tris-HCl buffer, pH 8.5, at 4°C. After sonication with an ultrasonic irradiator (VC 505, Sonics & Materials, USA), the cell lysate was centrifuged to remove cell debris. The supernatant was heated at 60°C for 20 min to remove unnecessary proteins by heat denaturation, and subsequently centrifuged. The obtained supernatant was purified with a Q Sepharose anion exchange column (GE Healthcare, USA) with 50 mM Tris-HCl buffer, pH 8.5, at 4°C. Fer8 was eluted with 300 mM NaCl. The eluted solution was diluted with 50 mM Tris-HCl buffer, pH 8.5, at 4°C and subsequently purified with a HiTrap Q HP anion exchange column (GE Healthcare) with a 0–500 mM NaCl gradient using a fast protein liquid chromatography (FPLC) system (Biologic DuoFlow 10, Bio-Rad, USA), and the absorbance was monitored at 280 nm. Subsequently, Fer8 was purified by size exclusion chromatography (SEC, HiPrep 26/600 Sephacryl S300, GE Healthcare) using the FPLC system at 4°C with 50 mM Tris-HCl buffer, pH 8.5, containing 150 mM NaCl, and the absorbance was monitored at 280 nm. The molecular mass of Fer8 was confirmed by matrix-assisted laser desorption ionization-time of flight mass spectrometry (MALDI-TOF MS) (Autoflex II, Bruker, USA) using sinapinic acid as a matrix. The purified Fer8 solution was concentrated with an Amicon Ultra ultrafiltration tube (Merck Millipore, 100,000 NMWL) to desired Fer8 concentrations. The concentration of Fer8 was calculated from the absorbance at 280 nm using the absorption coefficient 1.4 × 10^4^ M^-1^cm^-1^ [48].

For hydrogel formation, purified Fer8 in 50 mM Tris-HCl buffer, pH 8.5, was adjusted to concentrations of 6, 12, and 24 mM (subunit concentration). A small volume of 1 M HCl was added to the Fer8 solution to adjust the pH to 0.5, 0.6, 0.7, and 0.8 for acid denaturation, and the same volume of 1 M NaOH was added for neutralization. The components of the apoferritin hydrogel were investigated by MALDI-TOF MS (Autoflex-II, Bruker).

### Properties of apoferritin hydrogel

The water content of the apoferritin hydrogel was investigated by comparing the weights of dried and reswelled hydrogels. The hydrogels were dried by incubation at 50°C in a drying oven for 3 h. The stability of the apoferritin hydrogel was investigated by incubation in 1 M HCl, 1 M NaOH, 2-mercaptoethanol (2-ME), methanol, 43 μM trypsin, and 6 M guanidine hydrochloride (Gdn-HCl) at room temperature. The thermal stability of the apoferritin hydrogel was investigated by incubating the hydrogel in pure water at 20–100°C for 30 min. After the apoferritin hydrogel was heated at 90°C for 30 min, the heat-decomposed solution of the hydrogel was investigated by SEC (Superdex 200 increase 10/300 GL, GE Healthcare) using the FPLC system (Biologic DuoFlow 10, Bio-Rad) with 50 mM potassium phosphate buffer, pH 7.0, at 4°C, and the absorbance was monitored at 280 nm. The heat-decomposed solution of the hydrogel was also investigated by MALDI-TOF MS (Autoflex II, Bruker) using sinapinic acid as a matrix.

### Circular dichroism spectroscopy

The secondary structures of the heat-decomposed apoferritin hydrogel were investigated by circular dichroism (CD) spectroscopy with a J-725 spectrometer (Jasco, Japan) using a 0.1-cm-path length quartz cell at 25°C with 50 mM potassium phosphate buffer, pH 7.0.

### Atomic force microscopy for Young’s modulus evaluation

The modulus of the apoferritin hydrogel was assessed using a laboratory-built atomic force microscope (AFM) operated in ultra-pure water. A cantilever used had the dimensions with 10-μm long, 2-μm wide, and 90-nm thick (AC-10, Olympus) and the typical spring constant of this cantilever was 0.1 N/m. An amorphous carbon pillar with the end radius of ~5 nm, which was determined by scanning-electron -microscope observation, was used as the AFM tip. Force-distance curves were measured on the hydrogel surface. The Young’s moduli were estimated by fitting the force curves with the Hertz model using the tip radius of 5 nm and Poisson’s ratio of 0.5. Finally, a statistical histogram with a bin size of 5 kPa was produced for the Young’s moduli obtained from 1267 curves and fitted with a Gaussian distribution.

### Transmission electron microscopy

The apoferritin hydrogel was suspended in pure water and fragmented by sonication with an ultrasonic irradiator (US cleaner, Asone, Japan). The fragmented apoferritin hydrogel suspension was spread onto a carbon-coated 200-mesh copper grid (1606, JEOL, Japan), and negatively stained with 5% (w/w) phosphotungstic acid, pH 7.0. Transmission electron microscopy (TEM) images of the apoferritin hydrogel were taken with a JEM-3100FEF microscope (JEOL) at 300 kV.

### Metal adsorption measurements

To investigate metal ion adsorption to the apoferritin hydrogel, the hydrogel was swelled in pure water and subsequently incubated in 20 mM Fe(III)Cl_3_, Co(II)Cl_2_, Co(II) acetate, Ni(II)Cl_2_, Ni(II) acetate, or Cu(II)Cl_2_ solution at room temperature. The soaked hydrogel was transferred to pure water and subsequently treated with a 100 mM ethylenediaminetetraacetic acid (EDTA) solution. The hydrogel was monitored with a stereomicroscope (SZX16, Olympus, Japan). To obtain the metal ion:apoferritin subunit ratio in the ion-adsorbed hydrogels, the weight of the swelled hydrogel was measured. Subsequently, Fe^3+^, Co^2+^, Ni^2+^, and Cu^2+^ ions were dosed to the hydrogel. After washing the metal ion-dosed hydrogels with pure water, the hydrogels were denatured with aqua regia and the quantities of the metal ions were determined with a P-6000 microwave-induced plasma mass spectrometer (Hitachi, Japan) at 25°C.

## Results and discussion

### Preparation and water content of apoferritin hydrogel

We found that Fer8 forms hydrogels at room temperature by a simple denaturation procedure with the addition of 1 M HCl and subsequent neutralization with the addition of 1 M NaOH. The hydrogel formed when the Fer8 concentration was higher than 12 mM (subunit unit) and the acid denaturation pH was lower than 0.6, but not when the Fer8 concentration was 6 mM (subunit unit) or the denaturation pH was 0.7. Ferritin subunits are reported to undergo aggregation below pH 0.8 [35], indicating that denaturation of the subunit is necessary for the hydrogel formation. Apoferritin does not fully recover its hollow spherical structure by increasing the pH from acidic to neutral [35]. These results suggest incomplete refolding of the ferritin subunits by the pH recover from acidic to neutral, causing incomplete interactions between subunits and formation of a hydrogel. Refolding at high protein concentrations may cause three-dimensional domain swapping between subunits, which has been detected for cytochrome *c* and other globular proteins [49, 50].

**Fig 1:**
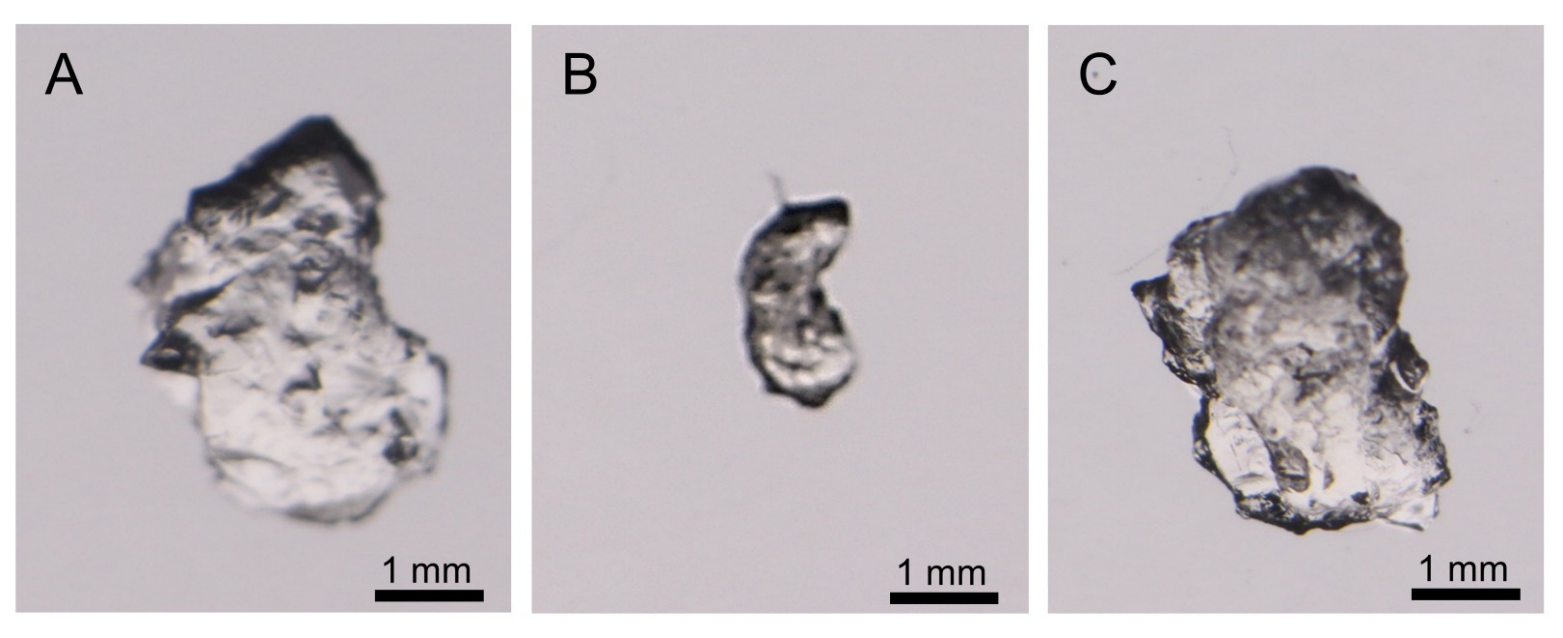
Microscopic images of apoferritin hydrogels. (A) Swelled, (B) dried, and (C) reswelled.

**Fig 2:**
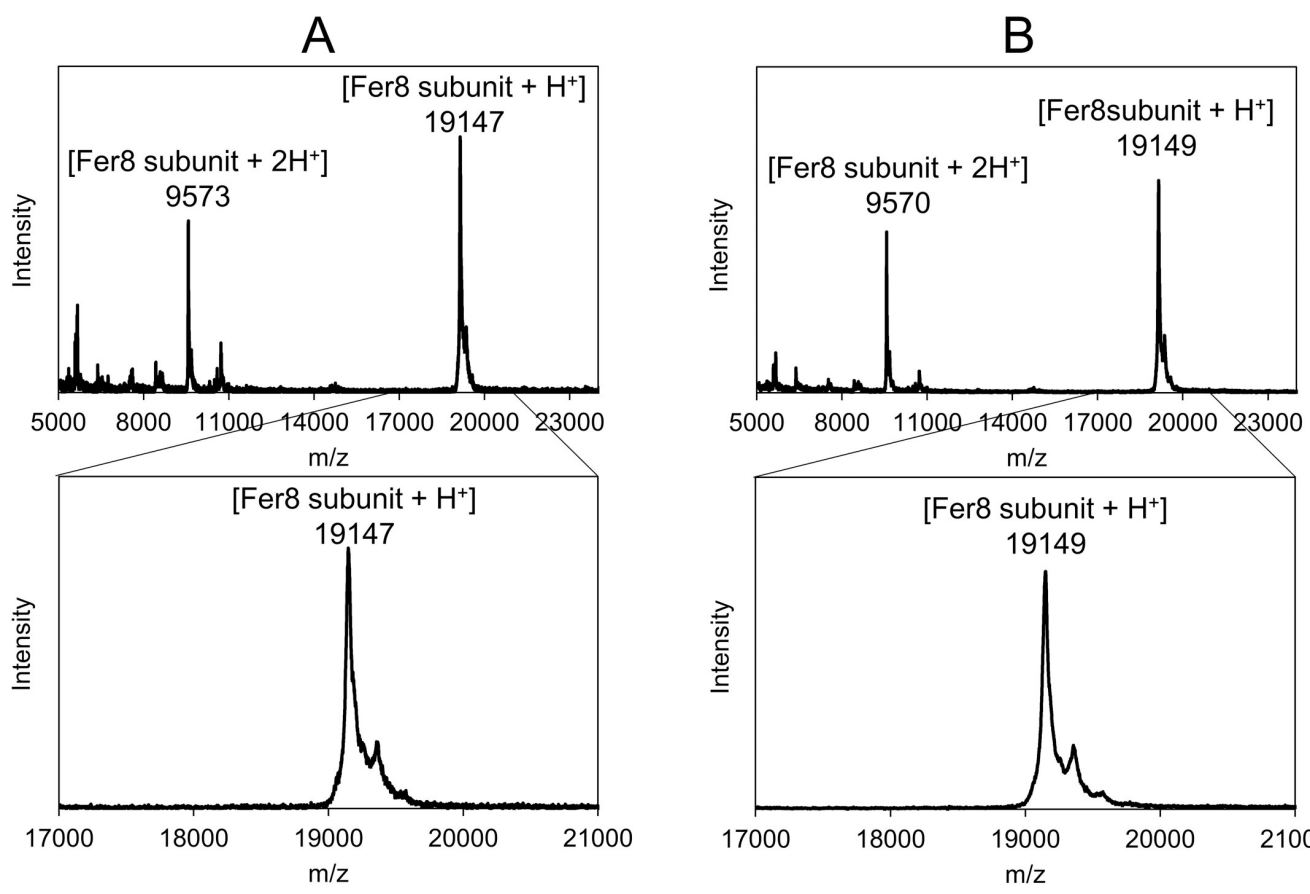
MALDI-TOF MS spectra of apoferritin hydrogel. (A) MALDI-TOF MS spectra of apoferritin hydrogel and (B) MALDI-TOF MS spectra of Fer8 solution after heating the apoferritin hydrogel at 90°C for 30 min. Wide range (top) and expanded range (m/z 17,000–21,000) (bottom). Sinapinic acid was used as a matrix.

The weights of swelled, dried, and reswelled apoferritin hydrogels were 12.1 ± 1.4, 2.3 ± 0.1, and 11.6 ± 1.0 mg, respectively (Fig 1), showing that the water content of the swelled hydrogel was approximately 80%. The weight of the reswelled hydrogel was almost the same as that before the drying process, indicating that the hydrogel is stable enough to repeat the drying and swelling procedure, although approximately 4% of the hydrogel may decompose by the procedure. The composition of the hydrogel was investigated by MALDI-TOF MS (Fig 2A); the peak at m/z = 19147, corresponding to the mass of the Fer8 subunit (theoretical value for [Fer8 subunit + H^+^] = 19148), was observed in the mass spectrum of the hydrogel, supporting the hypothesis that the hydrogel was constructed with Fer8 subunits.

### Properties of apoferritin hydrogel

**Fig 3:**
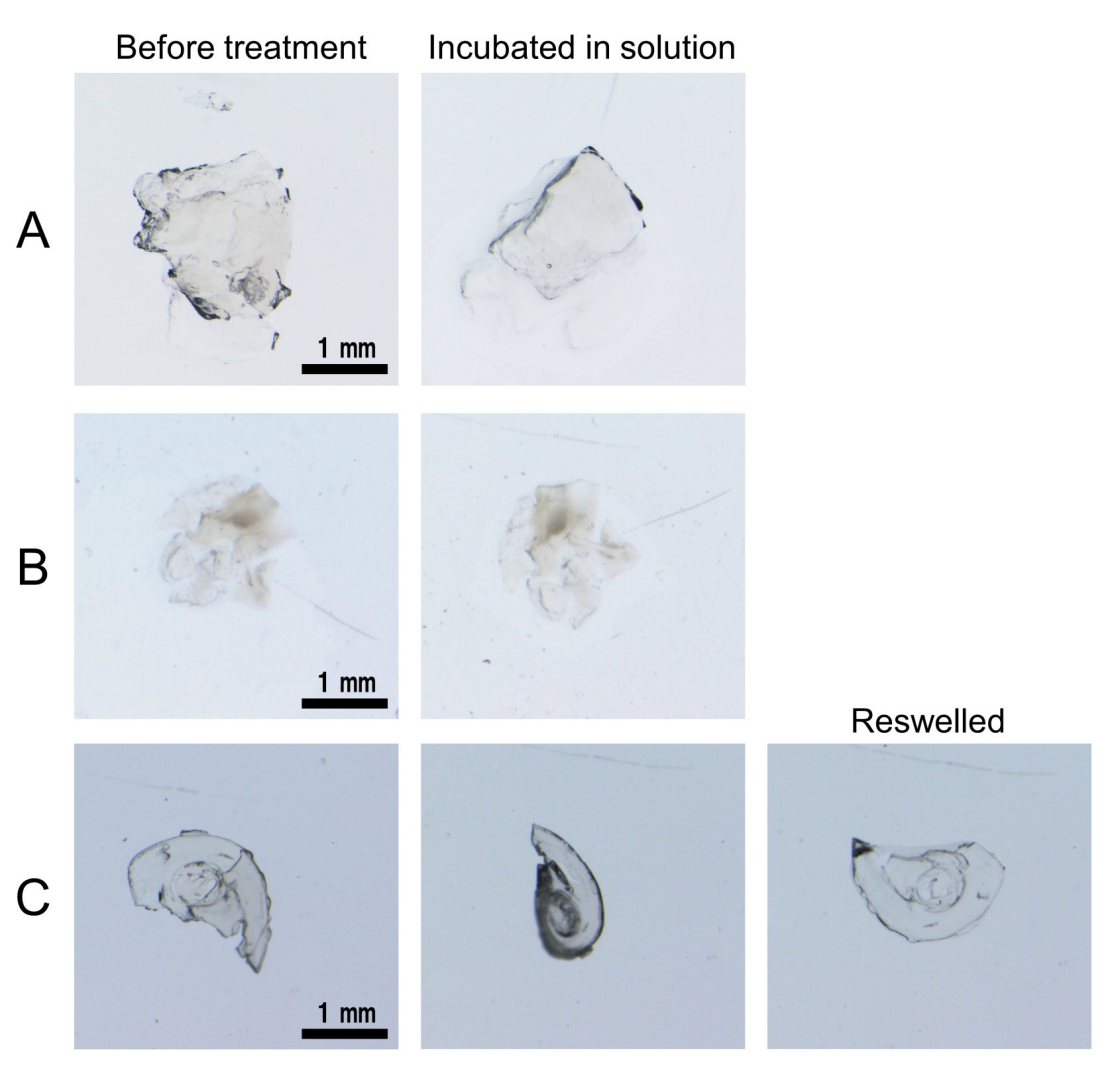
Microscopic images of apoferritin hydrogels before and after incubation in various solutions. (A) 1 M HCl and (B) 2-ME, and methanol. The hydrogels were incubated for1 h.

The stability of the apoferritin hydrogel was investigated by incubating the hydrogel at room temperature for 1 h under various conditions: 1 M HCl, 1 M NaOH, 2-ME, methanol, 43 μM trypsin, and 6 M Gdn-HCl. The shape of the hydrogel was not altered by incubation in 1 M HCl or 2-ME (Fig 3A and 3B), but decomposed in 1 M NaOH. The resistance of the hydrogel to 2-ME shows that the gel was not formed by disulfide bonds. The hydrogel was dehydrated by incubation in methanol, and the dehydrated hydrogel recovered its shape upon reswelling in pure water (Fig 3C). However, the hydrogel decomposed within 2 h by incubation in the presence of 43 μM trypsin or 6 M Gdn-HCl. These results are consistent with the hypothesis that the hydrogel was constructed from perturbed Fer8 subunits that decompose in the presence of proteases and unfold in the presence of high concentrations of denaturants.

**Fig 4:**
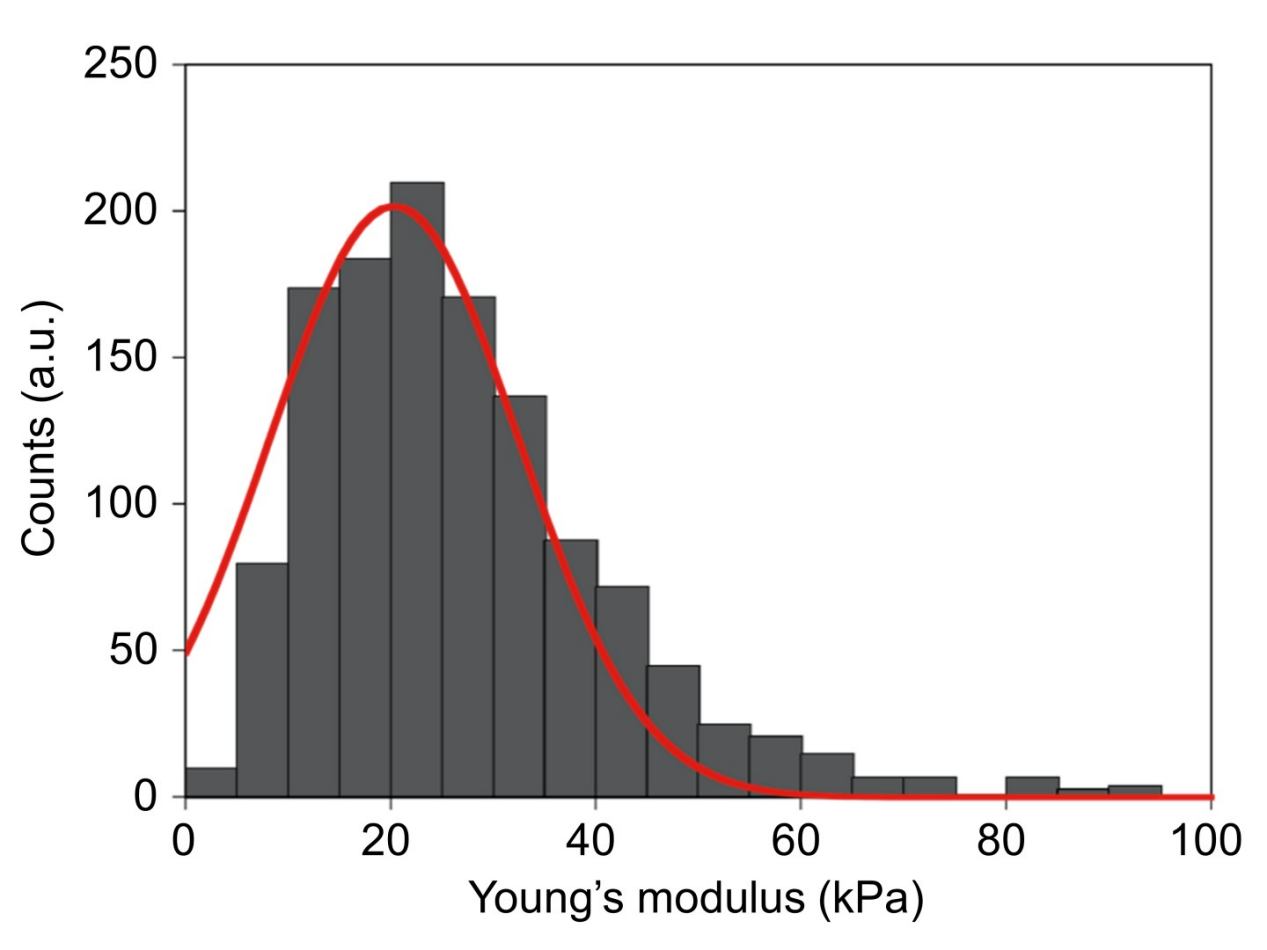
Young’s modulus of the swelled apoferritin hydrogel measured with AFM.

The apoferritin hydrogel was swelled in pure water and incubated for 30 min at 20, 40, 60, 80, and 100°C. The shape of the hydrogel did not change during incubation at 20–60°C, but slowly decomposed at 80–100°C. The thermostable character of the apoferritin hydrogel was similar to that of ferritin (denaturing temperature >80°C) [51], indicating that most of the three-dimensional structure of the Fer8 subunit and, in addition, the subunit–subunit interactions were maintained in the apoferritin hydrogel. The Young’s modulus of the hydrogel measured using AFM was 20.4 ± 12.1 kPa (Fig 4), showing that the hydrogel was relatively stiff compared to synthetic polyethylene glycol hydrogels [52, 53].

### Structure of apoferritin hydrogel

**Fig 5:**
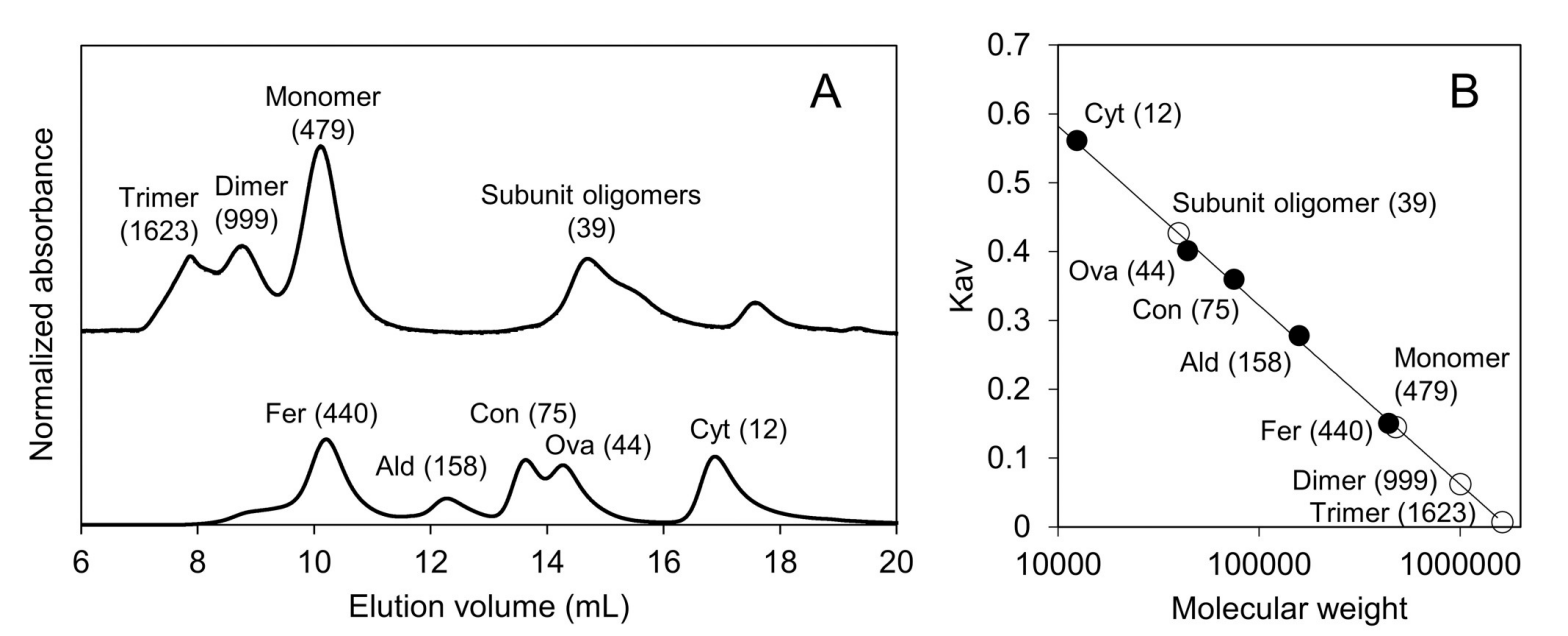
SEC analysis of the solution obtained after heating the apoferritin hydrogel at 90°C for 30 min. (A) Chromatograms of the solution obtained after heating the apoferritin hydrogel and of standard proteins (ferritin, 440 kDa; aldolase, 158 kDa; conalbumin, 75 kDa; ovalbumin, 44 kDa; cytochrome *c*, 12 kDa). (B) Standard curve obtained by least-square fitting of the partition coefficient (Kav) plots of standard proteins (closed circles). Peaks obtained in the chromatogram of the solution obtained by heating the apoferritin hydrogel are labeled with the molecular size (kDa) estimated by the standard curve. The plots of the apoferritin hydrogel after heating (open circles) are depicted with the estimated molecular weight (kDa).

**Fig 6:**
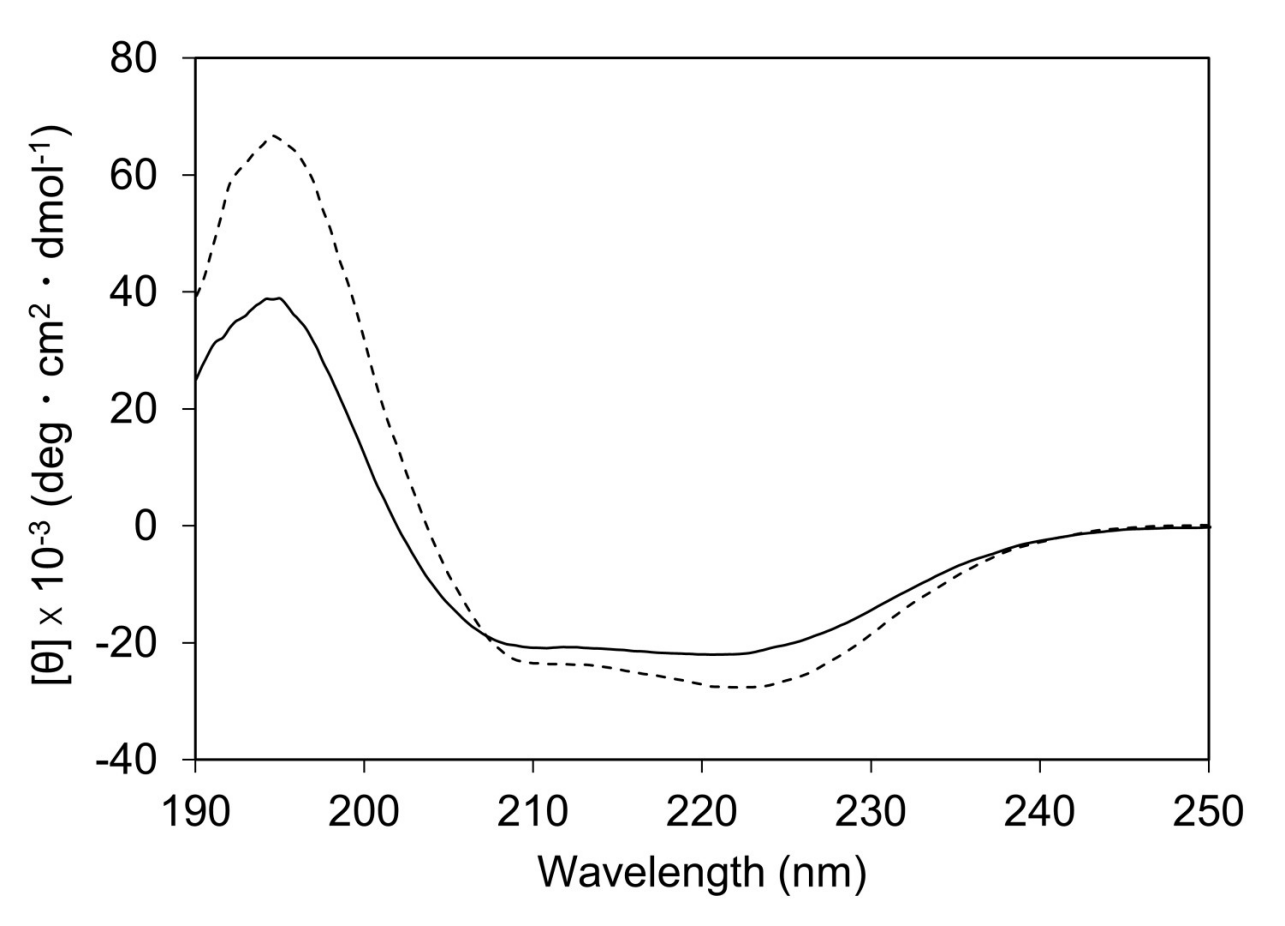
Circular dichroism spectra of Fer8 solution. Fer8 solution obtained after heating the apoferritin hydrogel at 90°C for 30 min (solid line) and purified Fer8 solution (broken line).

The heat (90°C)-decomposed solution of the hydrogel was analyzed with MALDI-TOF MS, and a peak corresponding to the mass of the Fer8 subunit (m/z = 19149) was observed in the spectrum (Fig 2B). In the SEC chromatogram of the heat-decomposed solution of the hydrogel, a peak corresponding to spherical ferritin with 24 subunits was observed (Fig 5). Additional peaks corresponding to the subunit and subunit oligomers of Fer8 were observed in the chromatogram, as were peaks corresponding to the dimer and trimer of spherical ferritin. The secondary structures of the heat-decomposed hydrogel were investigated with CD spectroscopy (Fig 6). The intensity of two negative peaks at 209 and 222 nm of the heat-decomposed hydrogel decreased compared to those of Fer8. These results indicate that Fer8 was partially unfolded in the hydrogel and the partially unfolded ferritin subunits refold into a spherical ferritin by the heating at 90°C.

**Fig 7:**
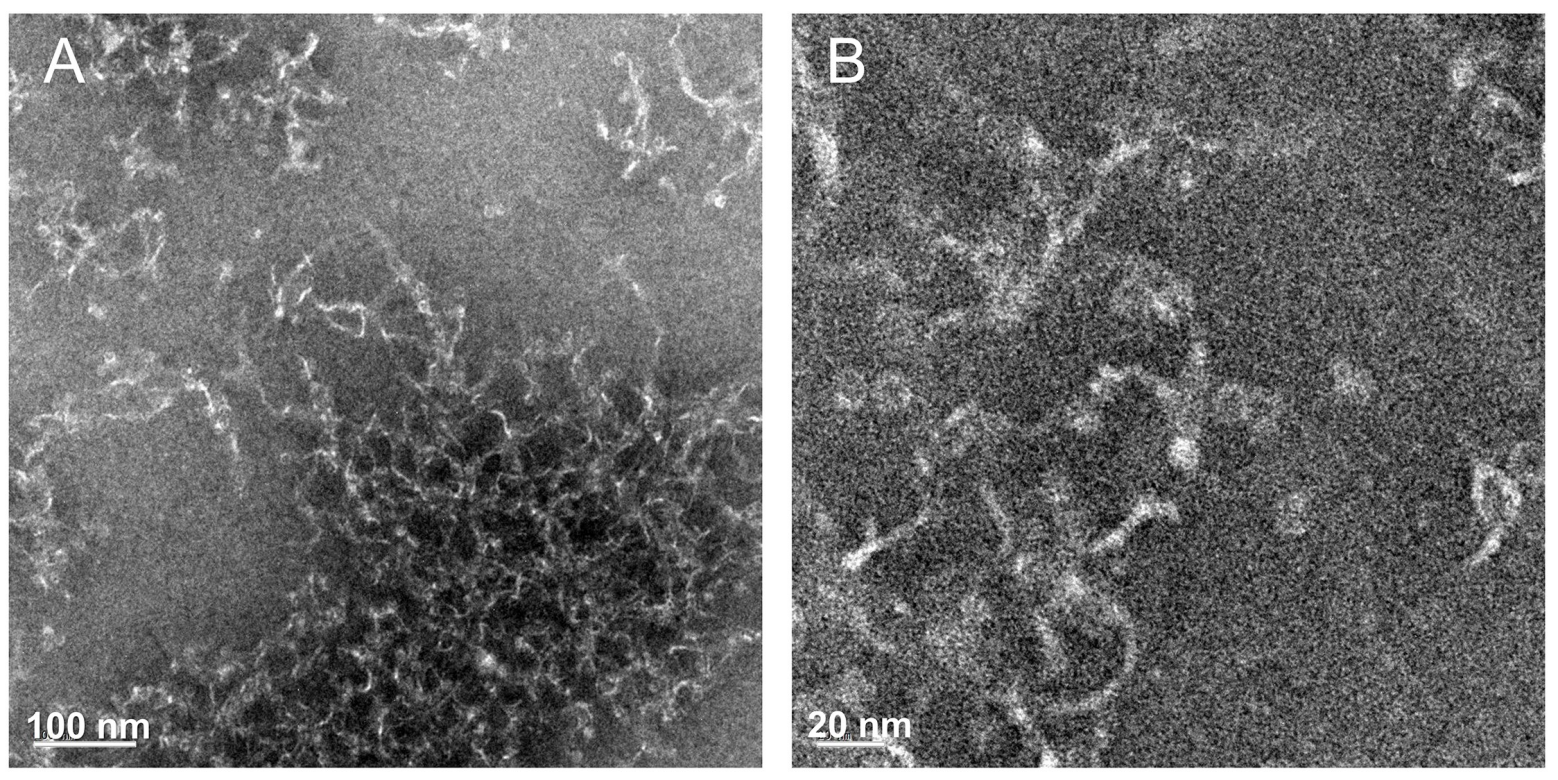
Negative-stain TEM images of apoferritin hydrogel. (A) Low and (B high resolution.

**Fig 8:**
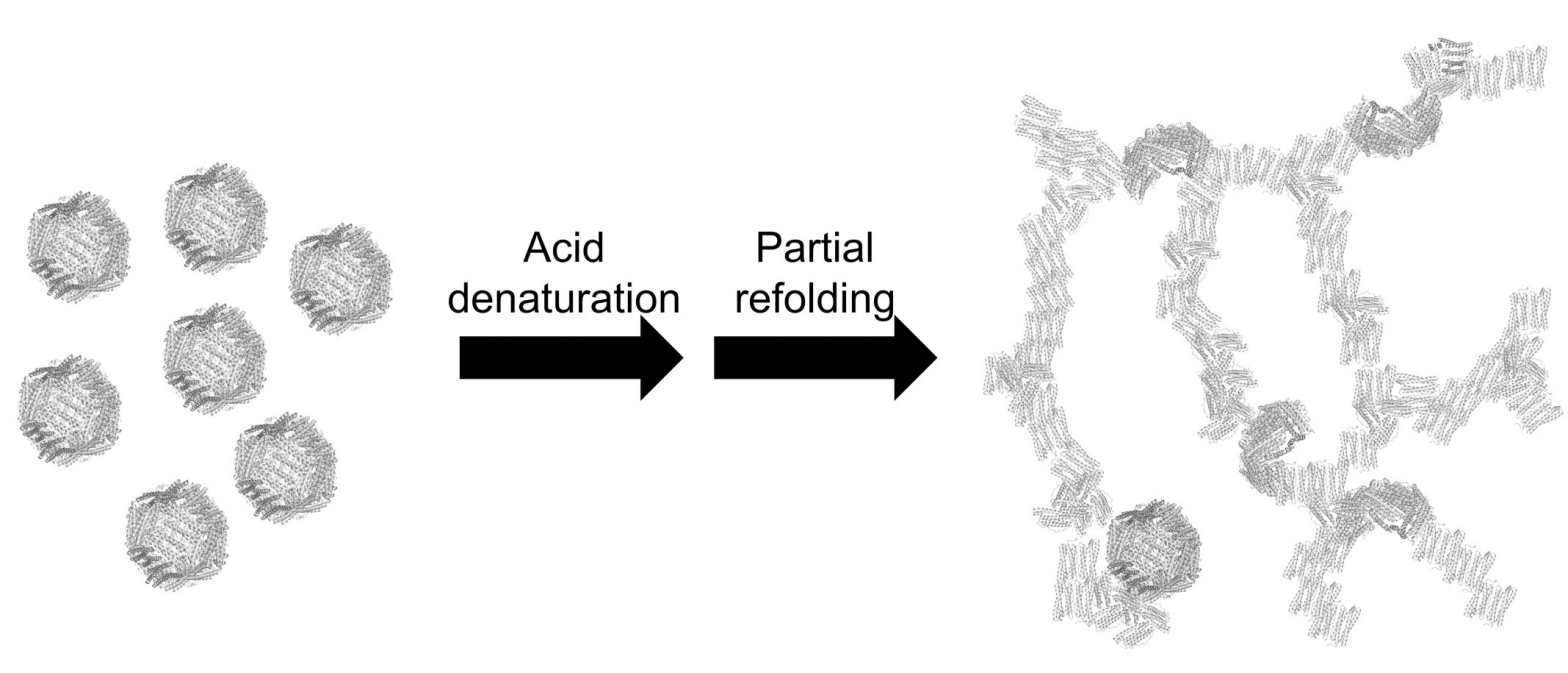
Schematic image of apoferritin hydrogel formation.

The microstructure of the apoferritin hydrogel was investigated with TEM, and network structures were observed (Fig 7). The high-resolution TEM images showed that the network structure contained spherical and fibrous structures (Fig 7B). The diameter of the spherical structure was 12 nm, which was close to that of spherical 24-mer ferritin. The diameter of the fibrous structure was relatively constant (~4 nm), corresponding to the width of two Fer8 subunits. Heat-set gels of globular proteins, such as β-lactoglobulin, lysozyme, and other proteins, contain partially folded structures [54]. Similarly, Fer8 exhibited partially disrupted fibrous structures in the apoferritin hydrogel (Fig 7). The partially unfolded Fer8 subunits may interact intermolecularly at various sites on their surfaces, forming the network structure of the hydrogel (Fig 8).

### Metal adsorption of apoferritin hydrogel

**Fig 9:**
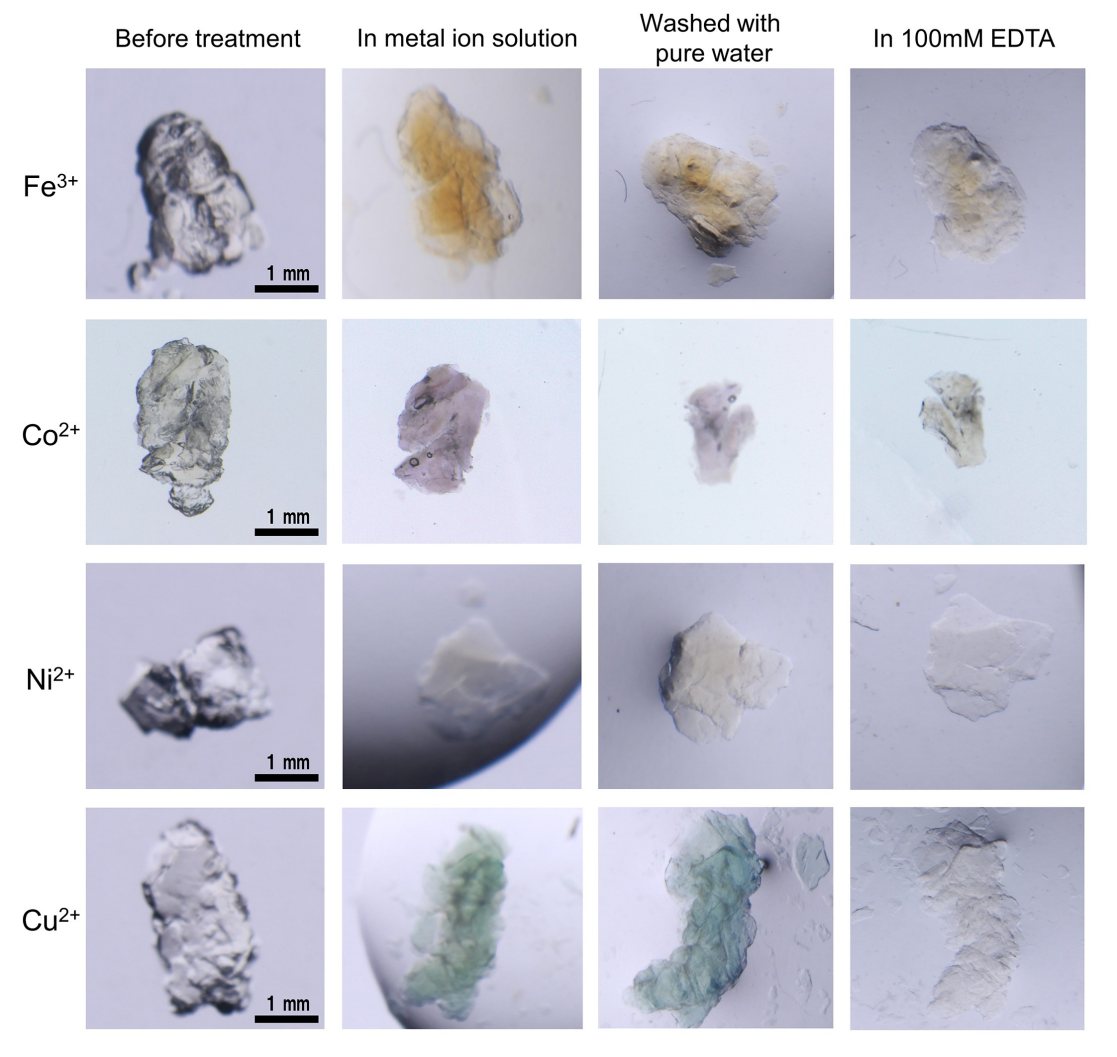
Metal adsorption of apoferritin hydrogel. Fe^3+^, Co^2+^, Ni^2+^, and Cu^2+^ ions are investigated. The apoferritin hydrogel was incubated sequentially in the presence of 100 mM metal ions for 1 h, in pure water for 3 h, and in 100 mM EDTA for 1 h. Fe(III)Cl_3_, Co(II) acetate, Ni(II)Cl_2_, and Cu(II)Cl_2_ solutions were used. The pictures of the hydrogel before the addition of the metal ions were taken under air, whereas other figures were taken under solutions.

Ferritin can store metal ions by metal–amino acid coordination [36–41]. Since Fer8 partially maintains its three-dimensional structure, we expected the apoferritin hydrogel to exhibit metal adsorption. The color of the hydrogel became yellow-brown upon incubation in the presence of Fe^3+^ ions for 1 h, indicating that the hydrogel adsorbs Fe^3+^ ions (Fig 9), similar to spherical ferritin. The yellow-brown color of the Fe^3+^-adsorbed hydrogel did not change upon washing the hydrogel with pure water but became pale upon incubation in the presence of 100 mM EDTA, suggesting that the Fe^3+^ ions were released from the hydrogel by EDTA coordination (Fig 9). Upon incubation in the presence of Co^2+^, Ni^2+^, or Cu^2+^ ions, the hydrogel became purple, pale yellow, or green, respectively, demonstrating that the apoferritin hydrogel may adsorb various metal ions, although the amount varied among ions. The adsorbed metal ion (μg) / apoferritin hydrogel (mg) values were 1.09, 0.55, 0.08, and 0.66 μg/mg for Fe^3+^, Co^2+^, Ni^2+^, and Cu^2+^ ions, respectively, according to the microwave-induced plasma mass analysis (Table 1). The Ni^2+^ ion did not bind strongly to the hydrogel, and more Fe^3+^ ions adsorbed to the apoferritin hydrogel than other metal ions, suggesting that the hydrogel keeps the iron storage characteristic of ferritin. However, the Fe^3+^ ion:apoferritin molar ratio was 1.9, indicating that only a small amount of ferritin was correctly folded. Ni^2+^ ions did not bind strongly to the apoferritin hydrogel when using either Ni(II)Cl_2_ or Ni(II) acetate, whereas Co^2+^ ions bound to it when using Co(II) acetate but not strongly when using Co(II)Cl_2_. These results show that the counter ions affect the metal binding ability of the hydrogel, presumably because the ligation of the counter ion, such as an acetate, to the metal ion may assist the metal ion binding to the amino acids in the hydrogel. The color of these metal ion-adsorbed hydrogels became pale upon incubation in the presence of 100 mM EDTA (Fig 9), showing that the apoferritin hydrogel adsorbs various metal ions and can release them in the presence of EDTA. These results support the hypothesis that the apoferritin hydrogel was constructed with ferritin subunits, maintaining their three-dimensional structures in metal binding.

**Table 1:** Amounts of metal ions adsorbed to the apoferritin hydrogel.

Metal ion	Adsorbed metal ion (μg) / Apoferritin hydrogel (mg)
Fe^3+^	1.09
Co^2+^	0.55
Ni^2+^	0.08
Cu^2+^	0.66

## Conclusion

Apoferritin forms a relatively stiff (Young’s modulus, 20.4 ± 12.1 kPa) hydrogel upon simple acid denaturation and subsequent neutralization. The hydrogel was stable up to 60°C and resistant to acidic pH conditions and a reducing agent (2-ME). The resistance of the hydrogel to 2-ME showed that the gel was not formed by disulfide bonds. The hydrogel formed a network of fibrils with relatively constant diameters (~4 nm), corresponding to the width of two Fer8 subunits, which indicated that the hydrogel was achieved by incomplete intermolecular interactions between Fer8 subunits. The hydrogel also exhibited metal ion adsorption/desorption properties, similar to spherical ferritin. These results show a new property of ferritin for future biomaterials.
